# Alkane degradation mechanism of *Mixta calida* HXX308 isolated from sediment of the Mariana Trench

**DOI:** 10.3389/fmicb.2025.1579612

**Published:** 2025-04-28

**Authors:** Yizi Yang, Xinxin He, Yulin Zhang, Xiao-Hua Zhang

**Affiliations:** ^1^Frontiers Science Center for Deep Ocean Multispheres and Earth System, and College of Marine Life Sciences, Ocean University of China, Qingdao, China; ^2^Laboratory for Marine Ecology and Environmental Science, Qingdao Marine Science and Technology Center, Qingdao, China; ^3^Key Laboratory of Evolution and Marine Biodiversity (Ministry of Education), and Institute of Evolution and Marine Biodiversity, Ocean University of China, Qingdao, China

**Keywords:** the Mariana Trench, *Mixta calida*, *Proteobacteria*, alkane degradation, comparative genomic analysis

## Abstract

The Challenger Deep of the Mariana Trench, which is the deepest site in the ocean, contains rich deposits of *n*-alkanes in its sediments. However, the alkane metabolic processes of the bacteria in this extreme environment were not well understood. In this study, we isolated a strain *Mixta calida* HXX308 (*Proteobacteria*) from sediment samples of the Challenger Deep (10,816 meters below sea level). HXX308 grows under pressures ranging from 0 to 40 MPa, with optimal growth at lower pressures. Additionally, it degrades approximately 20% of eicosane at both atmospheric pressure (0.1 MPa) and 20 MPa. Metabolic profiling indicated that HXX308 possesses a complete aerobic alkane metabolism pathway, along with nitrate reduction and sulfate reduction pathways, which support its adaptation to the trench’s anoxic environment. Comparative genomic studies showed that most strains in the genus *Mixta* contain the alkane-degrading gene *LadB*. Characterization of the *LadB* gene in HXX308 confirmed its role in the degradation of medium-to long-chain alkanes (C_18-36_). HXX308 is the first *Mixta* strain isolated from marine environment. Although this strain originated from the trench, its hydrocarbon metabolic characteristics are similar to those of cultures of terrestrial origin, suggesting that the alkanes in these sediments are likely from the terrestrial environment. Our study enhances the understanding of alkane-degrading in the phylum *Proteobacteria* and provides insights into the environmental adaptation of *M. calida* HXX308 in the Mariana Trench.

## 1 Introduction

The hadal zone (6,000–11,000 meters below sea level, mbsl), which is the deepest part of the ocean comprises trenches and submarine canyons, and accounts for approximately 45% of the total vertical depth of the ocean’s water mass (Jamieson et al., 2010). It is characterized by extreme environmental conditions, including complete darkness, high pressure (60 ~ 110 MPa), low temperature (1.0 ~ 2.5°C), low dissolved oxygen (156 ~ 172 μM), and relatively isolated topography, creating a unique and diverse biological community (Blankenship-Williams and Levin, 2009; Nunoura et al., 2015; Liu et al., 2023). Compared to the surrounding abyssal plains, the sedimentation in the hadal zone (>6,000 mbsl) is enhanced, which is attributed to seismically driven mass-wasting events (such as collapses and mudflows) along the slopes, and the funneling effect promoted by the “V-shaped” topography of the trench (Bao et al., 2018). Episodic downslope mass-transport events disrupt the continuous sedimentation at the deepest parts of the trench, introducing non-indigenous microorganisms that lead to the accumulation of organic carbon and alterations in redox conditions (Liu et al., 2024). These processes play a significant role in shaping the subseafloor community composition, and the erratic abundance and activity profiles with depth (Liu et al., 2024; Nunoura et al., 2015; Ichino et al., 2015).

These complex geological events, coupled with the unique environment of the trench, shaped unique microbial communities and their biogeochemical processes. It has been reported that sediments in various trenches are characterized by high organic matter content, abundant and active microbial communities, and rapid microbial carbon turnover, positioning hadal trenches as key sites for studying the degradation of deep-sea organic carbon (Wenzhöfer et al., 2016; Luo et al., 2018). Trench sediments are rich in hydrocarbons, with the average concentration of alkanes in surface sediments at a depth of approximately 10,900 m in the Mariana Trench being 2.3 μg/g dry weight, which supports the growth of hydrocarbon-degrading bacteria (Glud et al., 2013; Liu et al., 2019). Investigations have revealed that in the sediments at approximately 11,000 m of the Mariana Trench, the predominant aliphatic hydrocarbon-degrading genera including *Alcanivorax*, *Thalassolituus*, and *Oleibacter* (Liu et al., 2019). Additionally, the species *Venatorbacter* sp. C2-1 can grow with *n*-alkanes as the sole carbon source under *in situ* temperature (4°C) and pressure (58 MPa), demonstrating the adaptability of these bacteria to the extreme conditions of the hadal zone (Wang et al., 2023; Liu et al., 2023). These alkane-degrading bacteria in the Mariana Trench play multifaceted crucial roles in their habitat. Firstly, they are key participants in the carbon cycle, capable of converting complex organic carbon (like lignin, cellulose, and chitin) into simple inorganic carbon, thereby facilitating the carbon cycling within the marine ecosystem (Zhao et al., 2020; Tian et al., 2018). These bacteria reduce the accumulation of organic carbon in the sediments by degrading alkanes, maintaining the stability of the sedimentary environment. Moreover, their metabolic activities produce substances beneficial to other microorganisms, such as organic acids and amino acids, promoting the diversity and stability of microbial communities and sustaining the ecological balance of the hadal ecosystem (Wang et al., 2023). Additionally, during the process of alkane degradation, these bacteria actively participate in the nitrogen cycle, thereby influencing nitrogen transformations and cycling in the sediments. This participation is of significant importance for maintaining the balance of nitrogen cycling in the hadal environment (Yang et al., 2024; Huang et al., 2024). However, despite the progress made in research, the degradation potential and mechanisms of microorganisms in hadal sediments remain inadequately understood. Further in-depth studies of the functions and interactions of these microorganisms will aid in a better comprehension of the biogeochemical processes in trench ecosystems, as well as the adaptation and survival strategies of microorganisms in extreme environments (Arora and Panosyan, 2019).

In the sediments at the bottom of the Mariana Trench, *Proteobacteria* were identified as the dominant bacterial group, regardless of the method used, including pure culture techniques (Peoples et al., 2019), 16S rRNA gene amplicon sequencing (Cui et al., 2019) or metagenomic analyses (Chen et al., 2021; Liu et al., 2024). The genus *Mixta* belongs to the class *Gammaproteobacteria*, order *Enterobacterales*, and family *Erwiniaceae*. Species within this genus are characterized as facultatively anaerobic, motile, Gram-negative rods (Palmer et al., 2018). Till now, 14 species of genus *Mixta* were validly published,^1^ which were isolated from various environments, including plants, insects, food and the human body (Xia et al., 2020). The type species of genus *Mixta*, *M. calida* (type strain 1400/07^T^ = LMG 25383^T^ = DSM 22759^T^), was isolated from infant formula (Popp et al., 2010). Currently, research on various species of *Mixta* are focused primarily on their role as symbiotic bacteria in insects for plant disease control (Guz et al., 2020), and issues related to pathogenicity and food safety (Parra-Flores et al., 2022; Casale et al., 2023). However, the ecological functionality and importance of *Mixta* in natural environments remain significantly underestimated.

Total of 93 candidate hydrocarbon-degrading *Proteobacteria* strains were isolated from deep-sea trench sediments using a mixture of alkanes and diesel oil as the sole carbon source in our lab before. Among them, *M. calida* HXX308 is a representative strain of the genus *Mixta*, which was isolated from a marine environment for the first time and has not been previously reported to possess hydrocarbon-degrading capabilities. To explore the metabolic characteristics of strain HXX308, including its hydrocarbon degradation mechanisms and adaptation strategies to high-pressure environments, this study further investigated its metabolic network and hadal environment adaptation mechanisms based on high-pressure cultivation experiments, comparative genomic analysis, and heterologous characterization of the key hydrocarbon oxidation gene *LadB*. Our research provides a new perspective on how bacteria participate in the degradation of hydrocarbons to drive the material metabolic cycle in hadal environments.

## 2 Materials and methods

### 2.1 Sampling

A sediment core sample MT20-750 was collected from the Mariana Trench (11°19.904′N, 142°12.083′E) at depth of 10,816 m by the research vessel “*Dongfanghong 3*” in July 2020, as described in Liu et al. (2024). The sediment core, approximately 750 cm in length, is currently the deepest and longest sediment sample retrieved from the Challenger Deep. Utilizing mixed alkanes and petroleum diesel as the sole carbon sources, 93 cultures of potential hydrocarbon-degrading bacteria were isolated, belonging to 17 genera, predominantly from the class *Gammaproteobacteria*, followed by *Alphaproteobacteria* (Supplementary Figure S1). One representative strain from each of the 17 genera (Supplementary Figure S1) was selected to determine alkane degradation rates, with *Alcanivorax venustensis* ZYF844 used as the positive control (Supplementary Figure S2). The results showed that although *M. calida* HXX308 does not have as high degradation activity as some obligate alkane-degrading bacteria, it does exhibit hydrocarbon-degrading ability. Moreover, this strain is the first reported marine-derived strain in its genus, while other strains are terrestrial origin. Therefore, this study systematically investigates its metabolic characteristics and environmental adaptation mechanisms.

### 2.2 Genome sequencing and assembly

*Mixta calida* HXX308 was cultured in marine 2216E liquid medium at 28°C, 170 rpm until it reached the logarithmic phase (OD₆₀₀nm = 0.4 ~ 0.6), which took ~18 h. Cells were harvested by centrifugation at 12,000 rpm for 10 min at 4°C, then washed three times with 0.85% (w/v) saline to remove residual medium and growth metabolites (Liu et al., 2019). The cells were snap-frozen in liquid nitrogen, and sent to Guangdong Magigene Biotechnology Co., Ltd. (Magigene) for whole genome sequencing by using the PacBio Sequel IIe platform. Genomic DNA extraction and purified was performed using a proprietary method by Magigene. The integrity and purity of the DNA were assessed using 1% agarose gel electrophoresis. DNA concentration and purity were measured using Qubit 4.0 (Thermo Fisher Scientific, Waltham, USA) and NanoDrop One (Thermo Fisher Scientific, Waltham, USA).

After qualification, the genomic DNA was fragmented and end-repaired using G-tubes to prepare the SMRTbell DNA template library according to the manufacturer’s instructions. The library quality was evaluated using Qubit 4.0 (Thermo Fisher Scientific, Waltham, USA), and the average fragment size was assessed using Agilent 4200 (Agilent, Santa Clara, CA, USA). SMRT sequencing was performed on the Pacific Biosciences Sequel IIe sequencer (PacBio, Menlo Park, USA) following the standard protocol. The sequencing coverage depth was approximately 360X to ensure comprehensive genome coverage.

Prodigal v2.6.3 (Hyatt et al., 2010) was employed for coding gene prediction. The cleaning and assembly process of the raw Whole Genome Sequencing (WGS) sequences involved several steps. Initially, raw sequencing data were filtered to remove low-quality reads, adapter sequences, and reads with a high proportion of ambiguous bases (N’s). Subsequently, the cleaned reads were assembled using the Canu assembler v2.2 (Koren et al., 2017), which is designed for long-read sequencing data and is capable of producing high-quality genome assemblies. The assembly was further polished using the Arrow software to correct errors and improve the accuracy of the assembled genome.

Gene function annotations were performed by conducting whole-genome Blast searches against the following databases: NR (Non-Redundant Protein Database), Swiss-Prot, GO (Gene Ontology), KEGG (Kyoto Encyclopedia of Genes and Genomes), and COG (Clusters of Orthologous Groups). Secretory protein predictions were made using SignalP, LipoP, TMHMM, and PSORTb. Based on the assembly results and the predicted coding genes, along with analyses of non-coding RNA, gene functional annotations were visualized using Circos software (Krzywinski et al., 2009) to generate a whole genome map.

### 2.3 Phylogenetic analysis, gene annotation and metabolic pathway prediction

Twenty-one reference genomes closely related to *M. calida* HXX308 were obtained from the NCBI database (Sharma, 2018). The 16S rRNA gene of HXX308 were aligned with those of downloaded strains belonging to the genus *Mixta*. Multiple sequence alignment and trimming were performed using Muscle in MAGE 11 (Tamura et al., 2021), and a phylogenetic tree was constructed using the maximum likelihood (ML) method, with confidence values represented by bootstrap values based on 1,000 iterations.

To further determine the taxonomic position of these strains, the sequences of single-copy core genes from the whole genomes were extracted and used to develop phylogenetic trees using ML method. Specifically, Prokka v1.4.6 (Seemann, 2014) was used to functional annotate the 22 genomes, followed by the identification of homologous gene families in all genomes using Orthofinder v2.5.5 (Emms and Kelly, 2019). After obtaining the single-copy gene sequences shared by all species, multiple sequence alignment and trimming were performed using MAFFT and trimAl (Katoh and Standley, 2013; Capella-Gutiérrez et al., 2009). The core genes were concatenated, and the phylogenetic tree was constructed using IQ-TREE software (Nguyen et al., 2015), with confidence values indicated by bootstrap values based on 1,000 iterations. Finally, the phylogenetic tree was polished using the online tool Chiplot (Xie et al., 2023).

Following the method of Khot et al. (2021) hydrocarbon-degrading genes were predicted by aligning the annotated protein files with a concatenated HMM sequence file, defining sequences with an e-value < 10^−50^ as potential hydrocarbon-degrading proteins (Khot et al., 2021). Functional annotation of carbohydrate-active enzymes (CAZymes) was performed using the dbCAN module integrated with the CAZy database (*E*-value <10^−30^) (Lombard et al., 2013). This approach systematically identified and classified CAZyme families based on Hidden Markov Models (HMMs) to ensure accurate functional assignment. Annotation of protease families was conducted using the MEROPS database (E-value <10^−5^) (Rawlings et al., 2018), which provides comprehensive classification and functional information for peptidases. Additionally, the genomes were uploaded to the KEGG database^2^ for classification and annotation of metabolic pathways (Kanehisa et al., 2016), and a schematic of the metabolic pathways was created using Adobe Illustrator.

### 2.4 Bacterial cultivation and *n*-alkane degradation

To assess the *n*-alkanes degradation ability of *M. calida* HXX308 under high pressure conditions, ORN7a inorganic salt medium was prepared (details in Supplementary Table S1), distributed in air-tight syringes, and inoculated with freshly grown HXX308. Each syringe (ultraviolet disinfection for 30 min before use) was supplemented with 5 mL ORN7a medium with 1 mg of *n*-eicosane as the sole carbon source. These disposable plastic syringes were then placed in a stainless steel high-pressure vessel (380 mL, maximum pressure 60 MPa; Nantong Feiyu Oil Science and Technology Exploitation, China). Pressure was applied using water with a manual pump. Cultivation was conducted at room temperature for 30 days under four pressure conditions: 0, 20, 40 and 60 MPa.

Once the incubation was terminated, residual *n*-alkanes were extracted from the culture via ultrasonic extraction for three times with dichloromethane (DCM). The *n*-alkane content was analyzed using an Agilent 8860GC System detector, with an HP-5 chromatographic column (30 m × 320 μm × 0.25 μm) and helium as the carrier gas at a flow rate of 1.9775 mL/min. The temperature program was set with an initial temperature of 60°C, held for 1 min, and then increased to 280°C at a rate of 5°C/min, maintained for 25 min. A Flame Ionization Detector (FID) detector was used. A 10 μL syringe was used for injection, with 2 μL injected each time. *n*-Alkanes with different chain lengths present in the samples were determined by comparing their retention times with standards (Sigma), which included *n*-alkanes with chain lengths ranging from C_8_ to C_38_. The retention times of these standards were used to identify the specific *n*-alkanes in the samples. Concentrations were calculated based on their chromatographic responses relative to the internal standard deuterated n-tetracosane (D-24; Sigma), which was added to each sample to correct for any variations in sample injection volume and instrument response. All glassware used in this process was heated at 450°C for 6 h to remove organic contaminants.

To investigate the growth of *M. calida* HXX308 after cultivation under high pressure, a high-pressure point plate experiment was conducted. Before and after high-pressure cultivation, bacterial suspensions were serially diluted, and 10 μL amounts were spotted onto marine 2216E agar plates. These were incubated in a constant temperature chamber at 28°C for 24 h, and bacterial growth was subsequently observed.

### 2.5 Construction of the recombinant expression plasmid of the alkane degrading gene

The *n-*alkane oxidation gene *LadB* was cloned from *M. calida* HXX308, and the recombinant expression plasmid pRSET-A-*LadB* (Supplementary Figure S3) was constructed following the method of Wang et al. (2018). Briefly, the *LadB* gene sequence KGOCCCKL_01363, along with the two required electron transport proteins KGOCCCKL_03163 (Flavodoxin/ferredoxin-NADP reductase) and KGOCCCKL_04039 (2Fe-2S ferredoxin), were inserted into the expression plasmid pRSET-A (Bomaide Biological, Beijing). Each gene, including *LadB*, and two electron transport proteins, was equipped with a promoter and ribosome binding site (RBS) in its upstream region. The recombinant plasmid used specific primers for the *LadB* gene: *LadB*-F: 5′-ATGGCATTATCC GTATTC-3′; *LadB*-R: 5′-GCTTTGCGAGACTTTCTG-3′. The amplification products were analyzed by running agarose gel electrophoresis, and the presence of clear bands indicated successful transformation. The recombinant plasmid, verified for successful construction, was transferred into 100 μL of competent *Escherichia coli* BL21 cells using the heat shock method (42°C for 90 s) (Bomaide Biological, Beijing) (Froger and Hall, 2007). The transformed competent cells were then plated on LB agar plates (Haibo Biotech, Qingdao) containing ampicillin (50 μg/mL), and incubated at 37°C for ~24 h until colonies appeared. Single colonies were then transferred to 5 mL of fresh LB liquid medium supplemented with ampicillin (100 μg/mL) to prepare seed cultures for later use.

### 2.6 Verification of alkane degradation capability in recombinant strains

To verify the *n*-alkane degradation ability of the recombinant strain *E. coli* BL21-pRSET-A-*LadB*, 2 mL of overnight culture were inoculated into 150 mL of antibiotic-containing LB liquid medium, and incubated at 37°C with shaking at 170 rpm until the OD_600nm_ reached 0.5–0.6, which took ~3 h. Then, isopropyl-β-D-1-thiogalactopyranoside (IPTG) with the final concentration of 1 mM was added to the culture, with incubation for an additional 2 h to induce plasmid expression. Alkane degradation activity was then assessed. The induced expression system was centrifuged at 6000 rpm for 10 min at 4°C, and the supernatant was discarded. The retained cells were washed with M9 medium containing glucose and antibiotics (M9-glucose), and then resuspended in 90 mL of fresh M9-glucose medium with 90 μL of 50 mg/mL ampicillin. One milliliter of the resuspended cells was added to 10 mL of mixed alkanes (C_18_ and C_19_, 2.5 mg of each alkane per 10 mL) in ORN7a medium, and the mixture was incubated at 37°C with shaking at 170 rpm for 72 h. Additionally, two control groups included *E. coli* BL21-pRSET-A and *E. coli* BL21-pRSET-A-*Flavodoxin-2Fe-2S ferredoxin*, with un-inoculated blank medium as a negative control. Each group consisted of three replicates. Finally, the residual *n*-alkane concentration in the culture was extracted to determine the alkane degradation ability of the recombinant strain *E. coli* BL21-pRSET-A-*LadB*.

## 3 Results

### 3.1 The phylogenetic characteristics of *Mixta calida* HXX308

**Figure 1:**
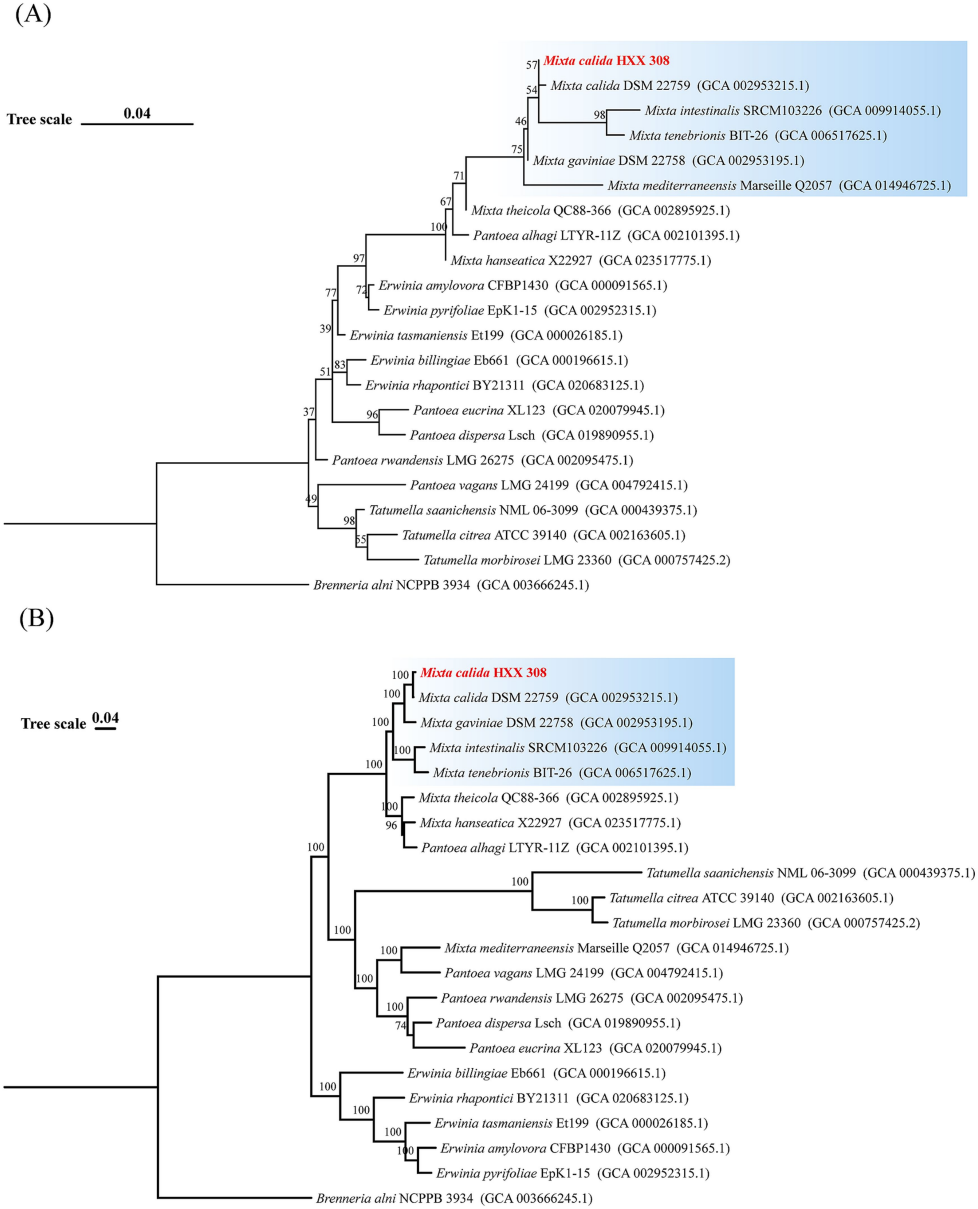
Phylogenetic tree of *M. calida* HXX308 and its related strains. **(A)** Phylogenetic tree constructed based on the 16S rRNA gene sequences of 22 strains. **(B)** Phylogenetic tree constructed based on the sequences of 1,226 single-copy core genes. The values at the nodes represent the bootstrap values based on 1,000 replicates. The scale bar indicates the substitution frequency per site.

The previous research in our laboratory isolated 93 potential hydrocarbon-degrading *Proteobacteria* from the deep-sea sediments using mixed alkanes and petroleum diesel as the sole carbon source. Among them, *Cereibacter* and *Mixta* have not been reported to have hydrocarbon-degrading capabilities. In this study, one representative strain was selected from each genus for alkane degradation capability testing (Supplementary Figure S1). It was found that different genera have different degradation preferences for chain length. Specifically, *Acinetobacter lwoffi* HXX052, *Erythrobacter nanhaiensis* HXX086, *Alcanivorax dieselolei* HXX443, *Tritonibacter mobilis* HXX101, and *Advenella kashmirensis* HXX265 showed weakened degradation capabilities with increasing alkane chain length. In contrast, other genera, including *M. calida* HXX308, exhibited stronger degradation capabilities with longer chain lengths. Moreover, this genus has not been reported to have hydrocarbon-degrading capabilities, so this paper conducts an in-depth exploration of its metabolic characteristics and environmental adaptation strategies. To clarify the evolutionary status of *M. calida* HXX308, we constructed 16S rRNA and core genome phylogenetic trees using sequences from *M. calida* HXX308 and 21 closely related strains. The 16S rRNA phylogenetic analysis revealed that *M. calida* HXX308 clustered with four species within the genus *Mixta*, namely *M. calida* 22759 to which it is most closely related, *M. intestinalis*, *M. tenebrioni*, and *M. gaviniae* (Figure 1A). The core genome phylogenetic tree, based on single-copy orthologous genes, yielded results that are essentially congruent with those of the 16S rRNA tree, further confirming that *M. calida* 22759 is the most closely related group to *M. calida* HXX308 (Figure 1B). These phylogenetic analyses provide a robust framework for understanding the evolutionary relationships within the Mixta clade, and highlight the taxonomic position of *M. calida* HXX308 within the phylum *Proteobacteria*, class *Gammaproteobacteria*, order *Enterobacterales*, and family *Erwiniaceae*.

### 3.2 Genomic characteristics of *Mixta calida* HXX308 and its reference strains

The circular genomic map of *M. calida* HXX308 (BioSample Accession: SAMN46867115) shows that it consists of a single chromosome containing 4,218,089 bp base pairs (Supplementary Tables S2–S4), with a GC content of 59.6% (Supplementary Figure S4). This genome includes 86 tRNA genes, eight 5S rRNA genes, seven 16S rRNA genes and seven 23S rRNA genes. Comparing the genomic characteristics of *M. calida* HXX308 with its reference strains (Table 1), it is evident that the HXX308 genome closely resembles that of the type strain of this genus (*M. calida* DSM22759), namely regarding genome size and GC content. However, the number of genes and encoded proteins in HXX308 are higher than those in *M. calida* DSM22759. Compared with other strains within the genus *Mixta*, HXX308 exhibits a smaller genome size (4.2 Mbp) and a higher GC percentage (56.9%) (Table 1). For example, the type strain *M. calida* DSM22759 has a genome size of 4.3 Mbp and a GC content of 57.0%. However, *M. calida* HXX308 was isolated from a marine environment, whereas other strains of *Mixta* were isolated from terrestrial habitats, including plants, insects, and food sources.

**Table 1:** The genomic characteristics of *M. calida* HXX308 with reference strains.

Species	Strains	Size (Mbp)	GC content (%)	Number of coding sequences	No. of genes	Number of contigs	Source
*Brenneria alni*	NCPPB 3934	4.1	51	3,814	4,127	132	*Alnus glutinosa*
*Erwinia amylovora*	CFPB1430	3.8	53.5	3,398	3,834	2	rosaceous plants
*E. billingiae*	Eb661	5.4	55	4,882	5,372	3	tree
*E. pyrifoliae*	EpK1/15	4.1	53.5	3,750	4,076	2	Apple twig
*E. rhapontici*	BY21311	5.2	54	4,706	5,165	2	*Apium graveolens*
*E. tasmaniensis*	Et1/99	4.1	53.5	3,709	4,068	6	flowers and bark
*Mixta calida*	HXX 308	4.2	56.9	4,261	4,367	1	Mariana Trench sediments
*M. calida*	DSM 22759	4.3	57	3,949	4,308	1	Powdered infant formula
*M. gaviniae*	DSM 22758	4.5	58	4,083	4,528	1	Powdered infant formula
*M. hanseatica*	X22927	4.4	54.5	4,121	4,445	5	male blood
*M. intestinalis*	SRCM103226	4.8	53.5	4,328	4,785	2	mealworm
*M. mediterraneensis*	Marseille Q2057	4.5	51	4,123	4,532	34	*Blomia tropicalis*
*M. tenebrionis*	BIT-26	4.7	56	4,149	4,655	73	*Tenebrio molitor*
*M. theicola*	QC88-366	4.3	54	3,987	4,291	72	black tea
*Pantoea alhagi*	LTYR-11Z	4.3	53.5	3,986	4,316	2	*Alhagi sparsifolia* Shap.
*P. dispersa*	Lsch	4.9	57.5	4,445	4,885	3	drinking water
*P. eucrina*	XL123	3.8	56.5	3,555	3,840	3	cucumber rhizosphere
*P. rwandensis*	LMG 26275	5.8	52.5	4,986	5,775	69	*Eucalyptus*
*P. vagans*	LMG 24199	4.8	55.5	4,333	4,790	3	*Eucalyptus*
*Tatumella citrea*	DSM 13699	4.5	49.5	4,080	4,491	2	mandarin orange
*T. morbirosei*	LMG 23360	4.4	50	4,085	4,413	6	pineapple
*T. saanichensis*	NML 06-3099	3.3	51.5	3,102	3,319	56	sputum

### 3.3 Metabolic network reconstruction of *Mixta calida* HXX308

**Figure 2:**
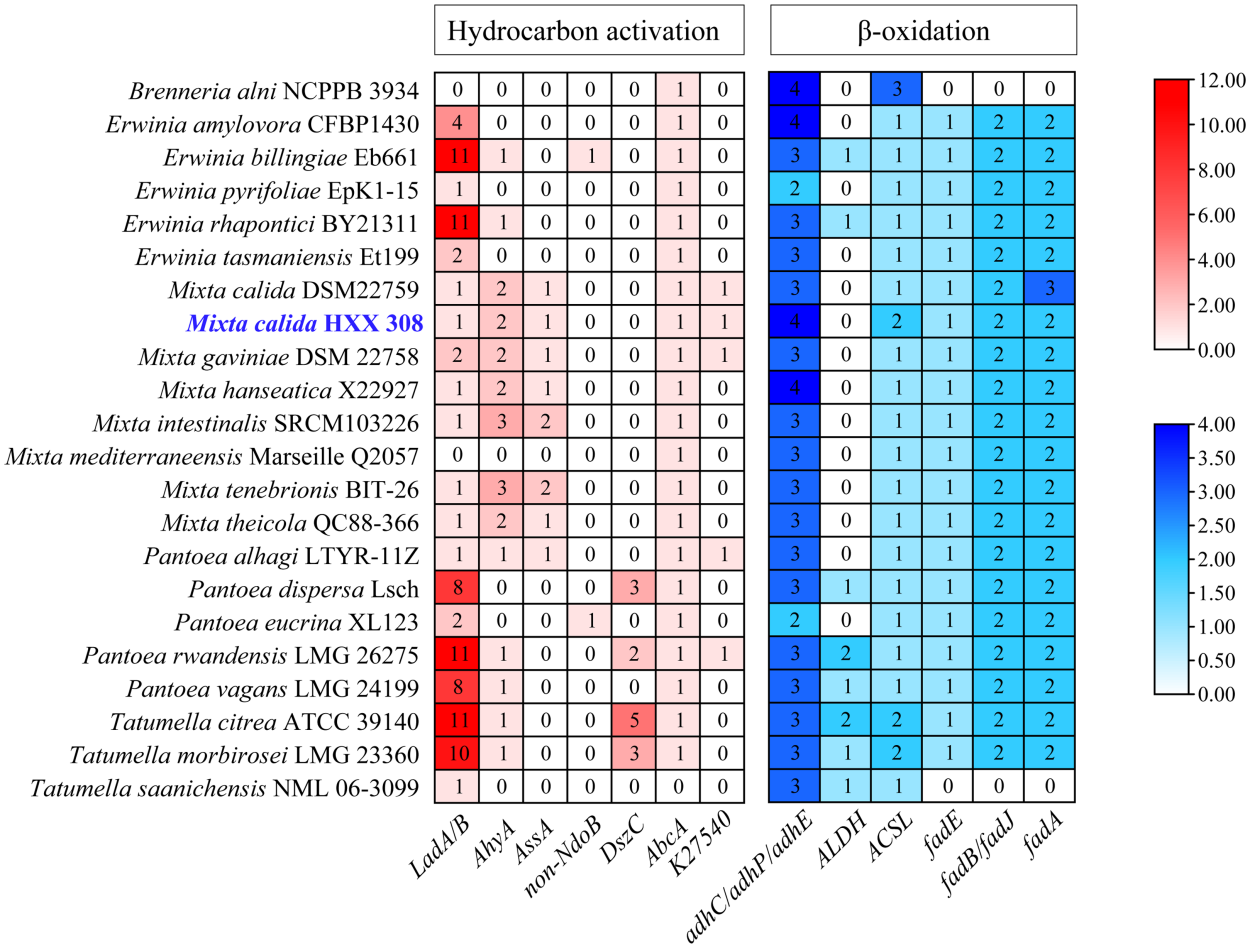
The hydrocarbon-degrading genes encoded by *M. calida* HXX308 and its reference strains. The depth of the legend color represents the gene copy number.

A comparison of alkane degradation related genes between HXX308 and reference strains revealed that the former encodes aerobic long-chain alkane degradation gene *LadB,* which is capable of degrading C_15-36_ *n*-alkanes. Additionally, it encoded genes, specifically *AhyA* or *AssA*, for anaerobic alkane degradation, and the genes, including *AbcA* or *K27540*, for anaerobic aromatic hydrocarbon-degrading (Figure 2). Our analysis indicates that HXX308 contains genes involved in aerobic alkane oxidation (*LadB*), anaerobic alkane oxidation (*AhyA* and *AssA*), and anaerobic aromatic hydrocarbon oxidation (*AbcA* and *K27540*). However, it is essential to clarify that while we have focused on key genes known to participate in these pathways, our analysis does not cover all possible genes that may also play a role. Therefore, we should avoid claiming that the pathway is ‘relatively complete’ without additional genetic evidence.

Instead, we can state that HXX308 has the genetic potential to engage in both aerobic and anaerobic alkane oxidation pathways, as well as in anaerobic aromatic hydrocarbon oxidation. This suggests a versatile metabolic capability. To ascertain whether HXX308 shares similar hydrocarbon-degrading genes with other strains, we conducted a comparative analysis using KEGG and CANT-HYD annotations, which involved comparing the presence and quantity of specific genes. Comparative analysis indicated that HXX308 shares identical hydrocarbon-degrading genes and β-oxidation genes with the model strain *M. calida* DSM 22759. In terms of aerobic alkane degradation, both HXX308 and the reference strains encode only the aerobic alkane degradation gene *LadB*. It is predicted that these strains can solely degrade long-chain alkanes under aerobic conditions.

**Figure 3:**
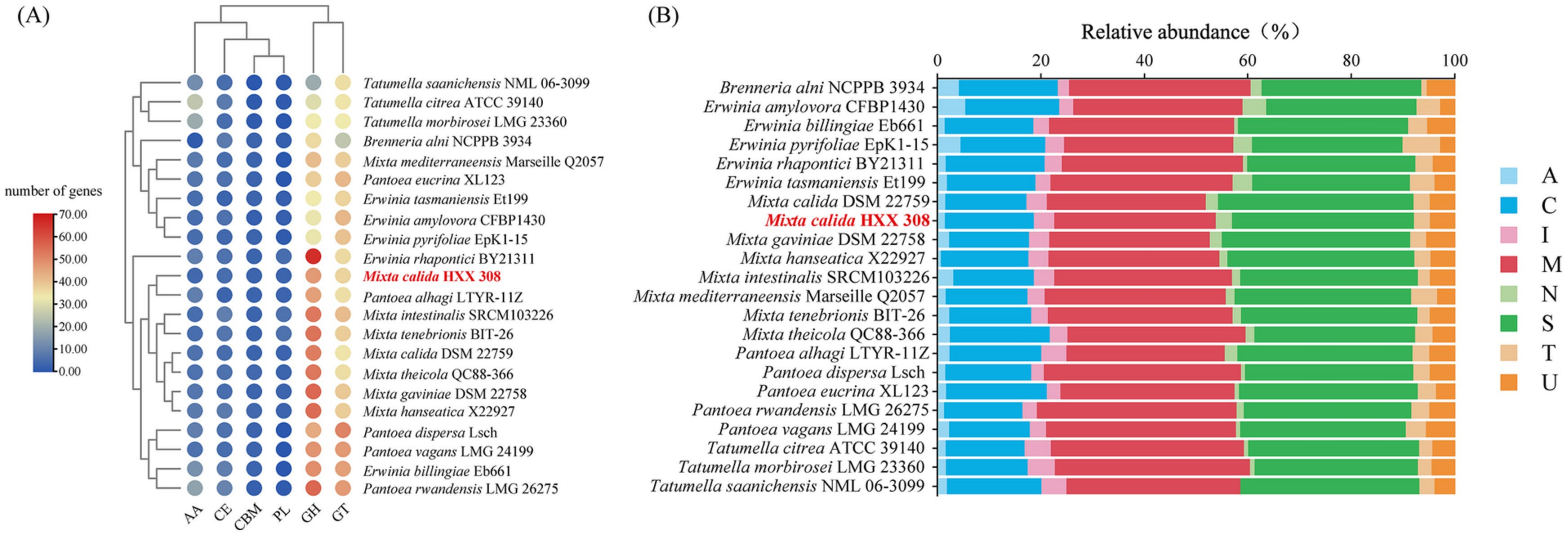
Comparative analysis of enzymes involved in carbohydrate metabolism and macromolecular protein utilization in *M. calida* HXX 308 and its reference strains. **(A)** Composition of carbohydrate-active enzymes in strain HXX 308 and its reference strains; **(B)** Functional categories of peptidases in strain HXX 308 and its reference strains.

**Figure 4:**
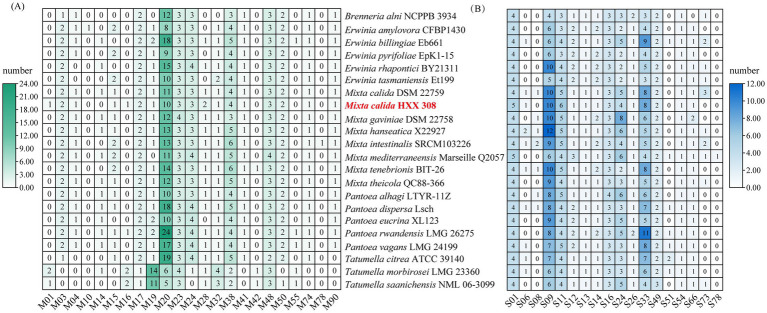
The absolute abundance of metalloprotease **(A)** and serine protease **(B)** encoded genes in the genomes of *M. calida* HXX 308 and its reference strains.

To further investigate the metabolic potential of these strains, particularly regarding carbohydrate degradation or synthesis and macromolecular proteins utilization, the CAZy and MEROPS databases were used to annotate CAZymes and peptidase families of these genomes. For carbohydrate metabolism, *Mixta* strains exhibited similar CAZymes copy numbers, with glycoside hydrolases (GHs) being the most abundant, followed by glycosyl transferases (GT), and the lowest copy numbers of carbohydrate-binding modules (CBM) and polysaccharide lyases (PL) (Figure 3A). *M. calida* HXX308 encodes 99 carbohydrate-active enzyme genes, including 47 GHs, 37 GTs, 5 CEs, 4 AAs, 3 CBMs, and 3 PLs (Figure 3A and Supplementary Table S6). This is similar to the type strain *M. calida* DSM 22759, indicating the similar potential of carbohydrate utilization. We annotated peptidases against the MEROPS database (Figure 3B and Supplementary Table S7) and, by comparing the number of peptidase families among different genera, found that the genus *Mixta* possesses a greater number of serine peptidases (S) and unclassified peptidases (U). For all genomes examined, the dominant peptidase families were serine proteases (S) and metalloproteases (M), with no representation of aspartic proteases (P) or glutamic acid proteases (G). Detailed analysis of metalloproteases and serine proteases across the genomes (Figures 4A,B and Supplementary Table S5) revealed that S09 (prolyl oligopeptidase) and M20 (glutamate carboxypeptidase) were the predominant families. In the genome of *M. calida* HXX308, the abundance of peptidases in family M20 was lower than that in other strains within the genus *Mixta*, whereas S11 (D-Ala-D-Ala carboxypeptidase A) was more abundant. Additionally, HXX308 possessed enzymes M1 (aminopeptidase N) and M16 (pitrilysin), which were absent in other reference strains.

**Figure 5:**
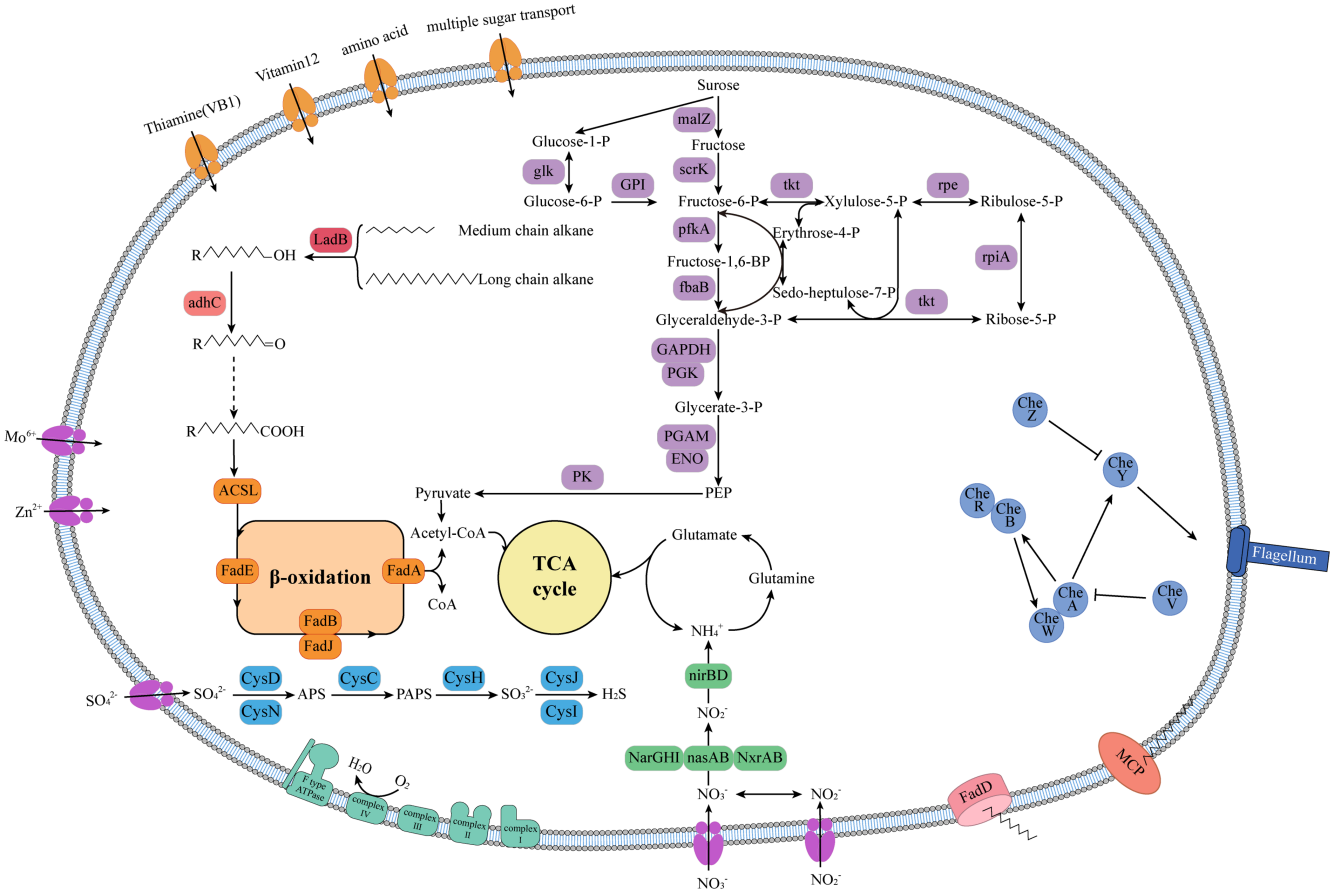
The metabolic pathway reconstruction of *M. calida* HXX 308.

To further elucidate the metabolic characteristics of HXX308, its metabolic pathway was reconstructed (Figure 5 and Supplementary Table S8), revealing the ability to utilize various carbon sources, including carbohydrates (fructose, sucrose, and glycerate), amino acids, oligopeptides, and phospholipids. This strain possesses a complete alkane metabolic pathway, enabling the entire process from alkane oxidation to acetyl-CoA, which is integrated into the TCA cycle. Additionally, HXX308 encodes 11 copies of the methyl-accepting chemotaxis protein (MCP), which aids in the bacterial perception of alkanes in the surrounding environment (Liu et al., 2015). MCP facilitates substrate degradation by attracting cells, whereas the transport of long-chain alkanes from the extracellular to the intracellular environment relies on outer membrane transport proteins, FadL (Zhou et al., 2017). Moreover, HXX308 possesses key genes that encode for assimilatory sulfate reduction and dissimilatory nitrate reduction, including nitrate reductase (*narGHI*) and nitrite reductase (*nirBD*). These pathways indicate the potential of HXX308 to utilize sulfate and nitrate found in sediments.

A comparative genomic analysis between HXX308 and DSM22759 (Table 2) revealed that the former harbors more genes related to oligopeptide transport, alkane chemotaxis, carbon fixation, and sugar metabolism. In contrast to HXX308, DSM22759 contains a higher number of genes with unknown functions.

**Table 2:** The list of KEGG Orthologs (KO) and gene numbers involved in certain metabolic processes in *M. calida* HXX308 and *M. calida* DSM22759.

Process	KO	Gene	Numbers	
			*M. calida* HXX308	*M. calida* DSM22759
ABC transporters	K15580	*oppA*, *mppA*; oligopeptide transport system substrate-binding protein	7	3
	K10823	*oppF*; oligopeptide transport system ATP-binding protein	2	1
Bacterial chemotaxis	K03406	*mcp*; methyl-accepting chemotaxis protein	11	8
	K03407	*cheA*; two-component system, chemotaxis family, sensor kinase CheA	3	2
	K03408	*cheW*; purine-binding chemotaxis protein CheW	2	2
	K03413	*cheY*; two-component system, chemotaxis family, chemotaxis protein CheY	2	2
	K03414	*cheZ*; chemotaxis protein CheZ	2	2
	K03412	*cheB*; two-component system, chemotaxis family, protein-glutamate methylesterase/glutaminase	2	2
	K03415	*cheV*; two-component system, chemotaxis family, chemotaxis protein CheV	1	1
	K00575	*cheR*; chemotaxis protein methyltransferase CheR	3	2
Carbon fixation	K00615	*tktA*, *tktB*; transketolase	8	5
	K01007	*pps*, *ppsA*; pyruvate, water dikinase	2	1
	K01626	*aroF*, *aroG*, *aroH*; 3-deoxy-7-phosphoheptulonate synthase	4	3
Glycolysis/Gluconeogenesis	K01810	GPI, *pgi*; glucose-6-phosphate isomerase	2	1
	K00873	PK, *pyk*; pyruvate kinase	2	3
	K01007	*pps*, *ppsA*; pyruvate, water dikinase	2	1
	K00163	*aceE*; pyruvate dehydrogenase E1 component	2	1
	K00627	DLAT, *aceF*, *pdhC*; pyruvate dehydrogenase E2 component (dihydrolipoamide acetyltransferase)	2	1
	K00121	*frmA*, ADH5, *adhC*; S-(hydroxymethyl)glutathione dehydrogenase/alcohol dehydrogenase	2	1
	K00138	*aldB*; aldehyde dehydrogenase	2	1
	K01835	*pgm*; phosphoglucomutase	2	1
	K01223	E3.2.1.86B, *bglA*; 6-phospho-beta-glucosidase	7	5
	K02753	*ascF*; beta-glucoside (arbutin/salicin/cellobiose) PTS system EIICB component	1	2
Others	K00558	DNMT1, *dcm*; DNA (cytosine-5)-methyltransferase 1	1	3
	K01185	lysozyme	1	5
	K07480	*insB*; insertion element IS1 protein InsB	16	19
	K06903	uncharacterized protein	0	3
	K06905	uncharacterized protein	0	3
	K06906	uncharacterized protein	0	4
	K06907	uncharacterized protein	0	4
	K06908	uncharacterized protein	0	3

### 3.4 The growth and alkane degradation capability of *Mixta calida* HXX308 under high pressure

**Figure 6:**
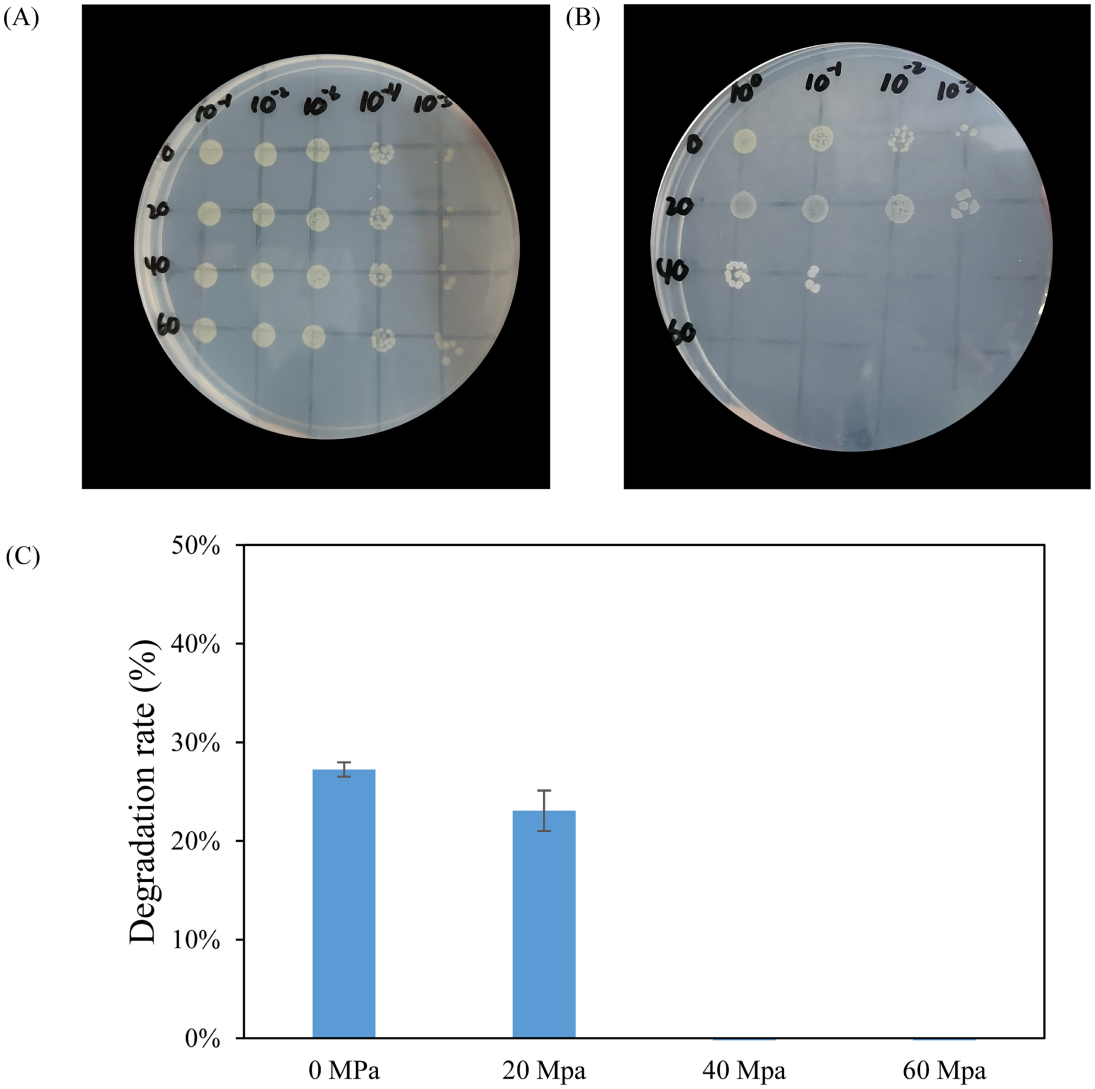
Verification of HXX308’s growth capacity under various pressures **(A)** Initial results from the high-pressure dot plate assay before high-pressure cultivation; **(B)** Results from the high-pressure dot plate assay following high-pressure cultivation; **(C)** Degradation rates after 30 days of incubation using C_20_ as the sole carbon source under various pressures. Error bars represent the standard deviation from triplicate measurements.

To assess the growth of HXX308 under different pressure conditions, a high-pressure processing was conducted at 0, 20, 40 and 60 MPa. The results indicated that HXX308 was able to grow under pressures ranging from 0 to 40 MPa, with growth progressively diminished as the pressure increased. Notably, no growth was observed after 30 days of incubation at 60 MPa (Figures 6A,B). The alkane degradation capacity of HXX308 under high pressure was also evaluated. Thus, HXX308 was able to degrade ~20% of C_20_ under both atmospheric (0.1 Mpa) and 20 MPa pressures (Figure 6C).

### 3.5 Alkanes degrading ability of *LadB* in *Mixta calida* HXX308

**Figure 7:**
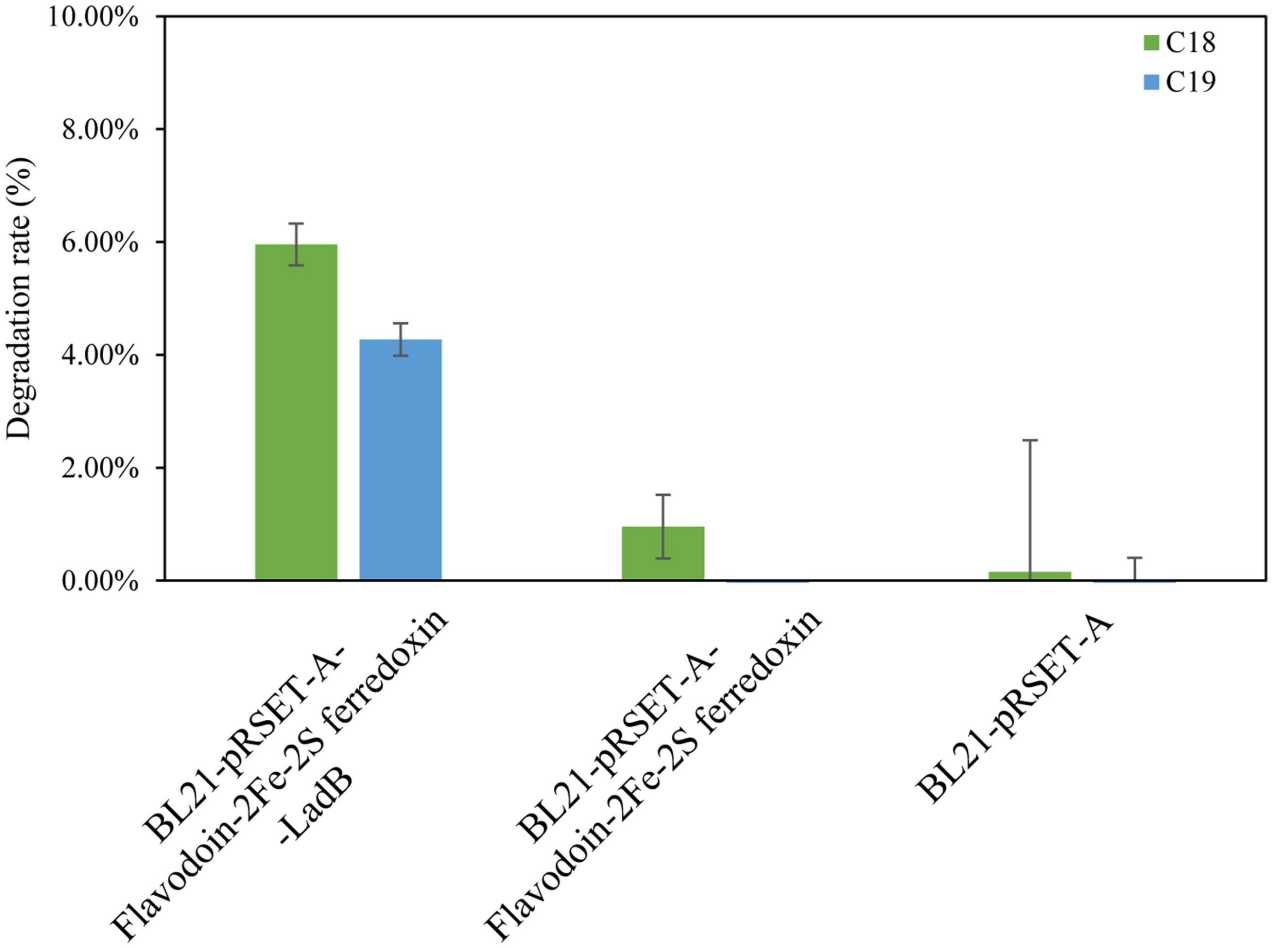
Cloning and expression of *LadB* gene in *M. calida* HXX308 and validation of alkane-degrading ability in the recombinant strain *E. coli* BL21-pRSET-A-Flavodoxin-2Fe-2S ferredoxin-*LadB*. Determination of the alkane degradation ability of the recombinant strains. The error bars are based on triplicate measurements.

Based on the annotation in the CANT-HYD database, HXX308 possesses the alkane-degrading gene *LadB*. The protein sequence analysis indicated that the sequence of *LadB* (KGOCCCKL_01363) from HXX308 showed the highest similarity (100%) to the FMNH2-dependent alkanesulfonate monooxygenase [*Mixta calida*] (NCBI GenBank ID: WP_312393135.1). LadB is an alkane monooxygenase that belongs to the LadA-type alkane monooxygenase family, and is capable of degrading long-chain alkanes ranging from C_11_ to C_32_ (Boonmak et al., 2014). However, the specific sequence and functional characteristics of *LadB* in HXX308 have not been extensively reported, suggesting that further investigation of this gene may provide novel insights into the mechanisms of alkane degradation. To verify the alkane-degrading activity of *LadB* in HXX308, the *LadB* gene was characterized. The recombinant expression strain *E. coli* BL21-pRSET-A-Flavodoxin-2Fe-2S ferredoxin-LadB was found to degrade 5.96% of C_18_ and 4.27% of C_19_ within 72 h (Figure 7 and Supplementary Figure S5), thereby confirming the alkane-degrading activity of the *LadB* gene in HXX308.

## 4 Discussion

### 4.1 The genus *Mixta* is generally characterized by its ability to degrade hydrocarbons

*Proteobacteria* is a dominant group in hadal trench sediments. In the Atacama Trench, *Proteobacteria* is one of the most abundant bacterial phyla, with its highest relative abundance observed in the oxic zone, particularly with *Gammaproteobacteria* comprising 20.9% of the total microbial community (Schauberger et al., 2021). Similarly, metagenomic sequencing and taxonomic analysis conducted by Zhang and Jing (2024) revealed that *Proteobacteria* is the dominant phylum in the surface sediments (0–4 cm) of the Challenger Deep in the Mariana Trench. *Proteobacteria* have been reported as dominant hydrocarbon-degrading taxa in hadal environments. In the Mariana Trench, the proportion of hydrocarbon-degrading bacteria at depths greater than 10,400 m is the deepest observed in any natural environment on Earth. These bacteria are primarily from the genera *Oleibacter*, *Thalassolituus* and *Alcanivorax*, which are known to consume aliphatic hydrocarbons (Liu et al., 2019; Teramoto et al., 2011; Wei et al., 2022; Kasai et al., 2002).

*Alcanivorax* is one of the most extensively studied genera for alkane degradation, particularly in marine environments, with its alkane metabolism mechanisms being thoroughly investigated (Gregson et al., 2019). However, the role of the genus *Mixta* in hydrocarbon-degrading in hadal environments remains unexplored. This study reports isolate *M. calida* HXX308, which was recovered from abyssal sediments at 487–490 cmbsf, and represents the first investigation of its alkane degradation processes.

The alkane degradation pathway is a complex regulatory network involving alkane sensing, chemotaxis, uptake, transport and metabolism (Rojo, 2009). In *Alcanivorax dieselolei* B5, alkane presence is sensed by OmpS, which activates a chemotaxis complex (comprising *mcp*, *cheR*, and *cheW*) to direct bacterial movement toward higher alkane concentrations (Wang and Shao, 2014). Genomic analysis of HXX308 revealed the presence of alkane chemotaxis protein genes (*mcp*) and a diverse set of chemotaxis-related genes (*cheR*, c*heA*, *cheB*, *cheW*, *cheV*, *cheY*, *cheZ*). Here, *cheR* and *cheB* regulate the methylation level of receptors to modulate *cheA* activity, which in turn phosphorylates *cheY* to regulate flagellar motion. *cheW* and *cheV* assist in signal transduction, whereas *cheZ* terminates signaling by dephosphorylating *cheY* (Xu et al., 2021).

In terms of alkane transport, *A. dieselolei* B5 utilizes selective outer membrane transport proteins (OmpT-1, OmpT-2, and OmpT-3) to transport alkanes of varying chain lengths into the cell (Wang and Shao, 2014). In contrast, HXX308 lacks OmpT proteins and instead employs FadL for the transport of medium-and long-chain alkanes across the outer membrane (van den Berg et al., 2004). While OmpT proteins can selectively transport short-, medium-and long-chain alkanes, FadL is specialized for medium-and long-chain alkanes (Liu et al., 2022).

Regarding alkane degradation, *A. dieselolei* B5 initiates the process via *AlmA*, which oxidizes alkanes to primary alcohols. These are subsequently oxidized to fatty acids by *AdhA* and *AldA*, followed by β-oxidation to generate acetyl-CoA, which enters the tricarboxylic acid cycle to produce carbon dioxide (Wang and Shao, 2014). HXX308 follows a different pathway, using *LadB* to oxidize alkanes to primary alcohols, which are then converted to fatty acids by *AdhC*. These fatty acids undergo β-oxidation to acetyl-CoA, ultimately entering the tricarboxylic acid cycle to generate carbon dioxide. In terms of substrate specificity, *AlmA* targets long-chain alkanes (C_26_–C_38_) (Chen et al., 2025), whereas *LadB* exhibits higher activity toward medium-and long-chain alkanes (C_15_–C_36_) (Feng et al., 2007; Wentzel et al., 2007).

In this study, we observed that *M. calida* HXX308 can grow under pressures ranging from 0 to 40 MPa in the laboratory, which does not encompass the pressure range of its native environment in the Mariana Trench (60–110 MPa). This discrepancy raises questions about the adaptability of HXX308 to the extreme conditions of the hadal zone. We further discuss possible reasons for this observation: (1) the lack of specific protective mechanisms in the laboratory setting, such as pressure-buffering substances or the synergistic effects of microbial communities; (2) the potential adaptation of HXX308 to lower pressure conditions during its cultivation in the laboratory; and (3) the possibility that the pressure in the native environment is the result of a long-term adaptation process that cannot be fully replicated in short-term experiments.

### 4.2 Adaptation of *Mixta calida* HXX308 to the trench environment

As a representative of extreme marine environments, hadal trenches are characterized by high pressure, low temperature and darkness (Wang et al., 2019). Microbes inhabiting these environments have evolved unique metabolic features to adapt to these extreme conditions. For example, in this study, *M. calida* HXX308 was found to possess genomic traits related to deep-sea adaptation, including a streamlined genome with a higher ratio of proteins to genes compared to other strains of *Mixta* (Liang et al., 2022). This streamlined genome facilitates adaptation to oligotrophic environments. Similar genome streamlining has been observed in *Parcubacteria* transitioning from near-surface soils to groundwater habitats (Chaudhari et al., 2024). Additionally, streamlined strains of *Bacillus subtilis* have been shown to exhibit high resistance to DNA-damaging agents (Dervyn et al., 2023).

Moreover, *M. calida* HXX308 has metabolic traits that enable it to adapt to hypoxic environments. Sediments in the Mariana Trench typically have lower dissolved oxygen content compared to open water, with oxygen concentrations decreasing with depth due to microbial respiration and organic matter mineralization (Li et al., 2019; Xiao et al., 2024). To survive in these low-oxygen conditions, microorganisms often adjust their metabolic pathways, utilizing alternative respiratory chain acceptors or anaerobic respiration. For example, *Pseudomonas alcaliphila* strain MBR uses an electrode as an electron donor for dissimilatory nitrate reduction to ammonium and denitrification (Su et al., 2012). Similarly, heterotrophic denitrifiers oxidize sulfide to mitigate toxicity and support complete denitrification, providing a competitive advantage in low-oxygen environments (Shao et al., 2025).

*M. calida* HXX308 possesses key genes that encode for the assimilatory sulfate reduction pathway and the F-type ATPase, indicating its ability to utilize sulfate reduction—a primary anaerobic respiration pathway—in hypoxic environments. The expression of *narGHI* under low-oxygen conditions further suggests that HXX308 may use nitrate as an electron acceptor, reducing it to nitrite and then to ammonium, consistent with the low-oxygen environment of Mariana Trench sediments (Mareen et al., 2021).

### 4.3 *Mixta calida* HXX308 possesses more genes adapted to the trench environment than the reference strain *Mixta calida* DSM22759

Currently, known strains of the genus *Mixta* have been isolated from terrestrial environments, such as plants, insects, food and the human body (Xia et al., 2020). However, this study isolated *M. calida* HXX308 from sediment at a depth of 10,816 m in the Mariana Trench, marking the first marine-sourced strain of the genus. Despite its origin in the trench, HXX308 exhibits hydrocarbon metabolic characteristics similar to those of the type species *M. calida* DSM22759.

Detailed analysis revealed that HXX308 possesses more genes encoding the oligopeptide transport system substrate-binding protein (OppA) compared to *M. calida* DSM22759 (Table 2). OppA is a key component of the oligopeptide transport system, responsible for peptide uptake and providing bacteria with nutrients and environmental status information (Monnet, 2003). In nutrient-poor environments, like the trench, the expression of OppA is upregulated to enhance nutrient acquisition (Urbanowski et al., 2000). This suggests that HXX308 has adapted to the oligotrophic conditions of the trench by increasing its capacity for peptide transport.

Additionally, HXX308 has more copies of genes including *tktA*, *aroG*, and *pps,* involved in aromatic compound synthesis. These genes are crucial for the production of 3-deoxy-D-arabinoheptulosonate-7-phosphate (DAHP), a key intermediate in aromatic compound biosynthesis (Patnaik et al., 1995; Vimala and Harinarayanan, 2016; Shaw et al., 2018). The increased presence of these genes likely enhances HXX308’s ability to fix carbon and survive in nutrient-limited environments.

In the glycolysis/gluconeogenesis pathway, HXX308 exhibits a greater number of related genes, particularly *bglA*. The enzyme encoded by *bglA*, 6-phospho-β-glucosidase, catalyzes the hydrolysis of 6-phospho-β-glucosides to produce glucose-6-phosphate, facilitating cellulose breakdown and sugar metabolism (Desai et al., 2010; Veldman et al., 2020). This suggests that HXX308 has a stronger capacity for sugar metabolism compared to terrestrial strains, such as *M. calida* DSM22759.

Overall, the increased number of genes related to peptide transport, aromatic compound synthesis, and sugar metabolism in HXX308 highlights its adaptation to the oligotrophic and extreme conditions of the Mariana Trench.

## 5 Conclusion

In this study, we investigated the alkane degradation *Proteobacteria* strain *M. calida* HXX308, which was isolated from deep-sea sediment in the Mariana Trench. High-pressure alkane degradation assays and high-pressure processing indicated that strain HXX308 could grow at pressures ranging from 0 to 40 MPa, with reduced growth at higher pressures. It could degrade approximately 20% of eicosane at atmospheric pressure (0.1 MPa) and 20 MPa. Phylogenetic analysis revealed that HXX308 is most closely related to *M. calida* DSM 22759. HXX308 is capable of extensively participating in the processes of alkane metabolism, and nitrogen and sulfur cycling within sediments. Compared to terrestrial strains of the *Mixta* genus, HXX308 exhibits a greater number of adaptations to the Hadal Trench environment. Specifically, it encodes a higher abundance of genes associated with peptide transport, aromatic compound synthesis and carbohydrate metabolism. Collectively, these genetic features enhance its capacity for nutrient acquisition and metabolic versatility in the hypoxic and oligotrophic conditions of the trench.

## Data Availability

The datasets presented in this study can be found in online repositories. The names of the repository/repositories and accession number(s) can be found below: https://www.ncbi.nlm.nih.gov/, SAMN46867115.

## Footnotes

^1^ https://lpsn.dsmz.de/species/mixta-calida

^2^ https://www.kegg.jp/ghostkoala/
