# Supplementary material for: Alkane degradation mechanism of Mixta calida HXX308 isolated from sediment of the Mariana Trench
Source: Front Microbiol. 2025 Apr 28;16:1579612. doi: 10.3389/fmicb.2025.1579612 (PMC12066429; doi:10.3389/fmicb.2025.1579612)
Supplement: Supplementary file 1 [file Data_Sheet_1.docx]

**Supplementary Materials**

**
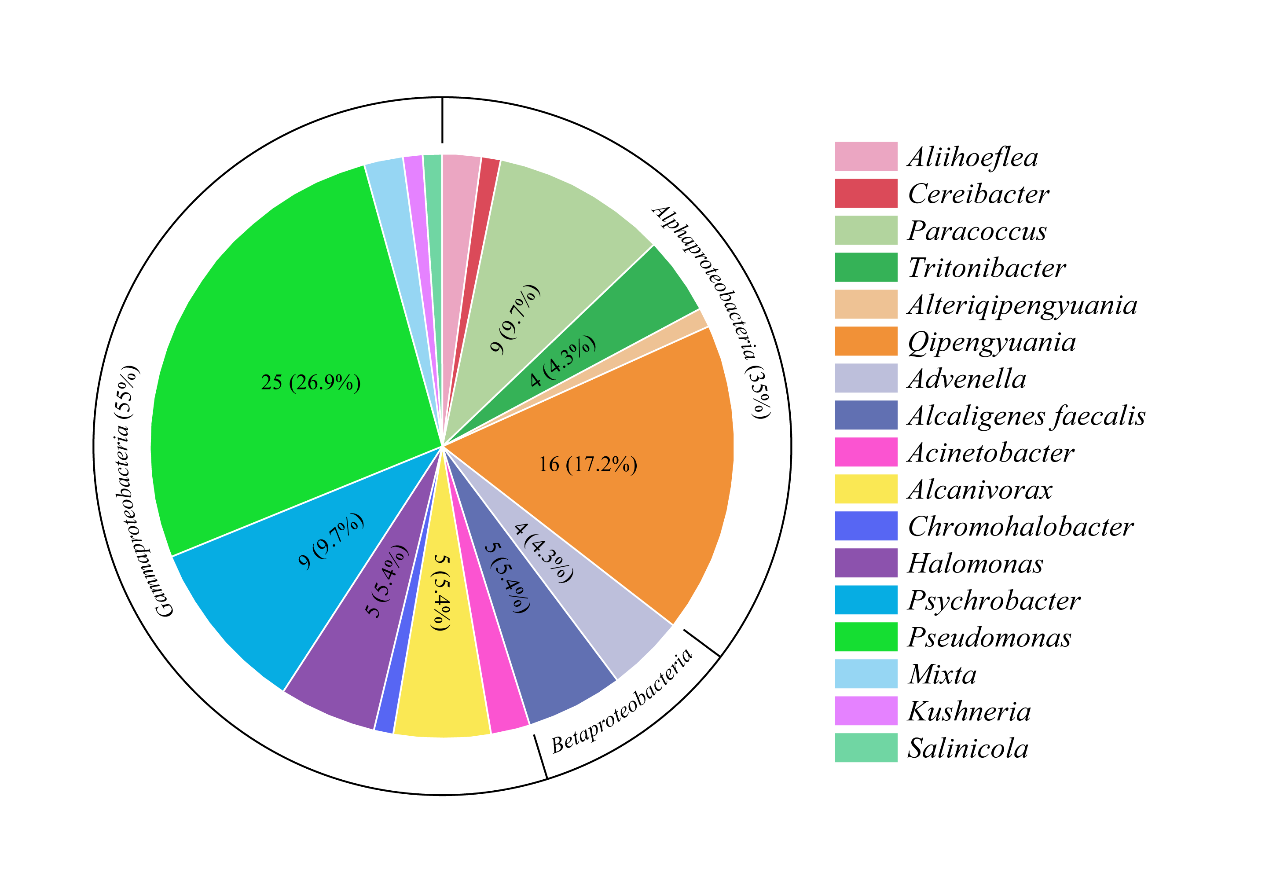
**

**Figure S1.** The classification results of *Proteobacteria* in Mariana Trench sediments at the genus level.

**
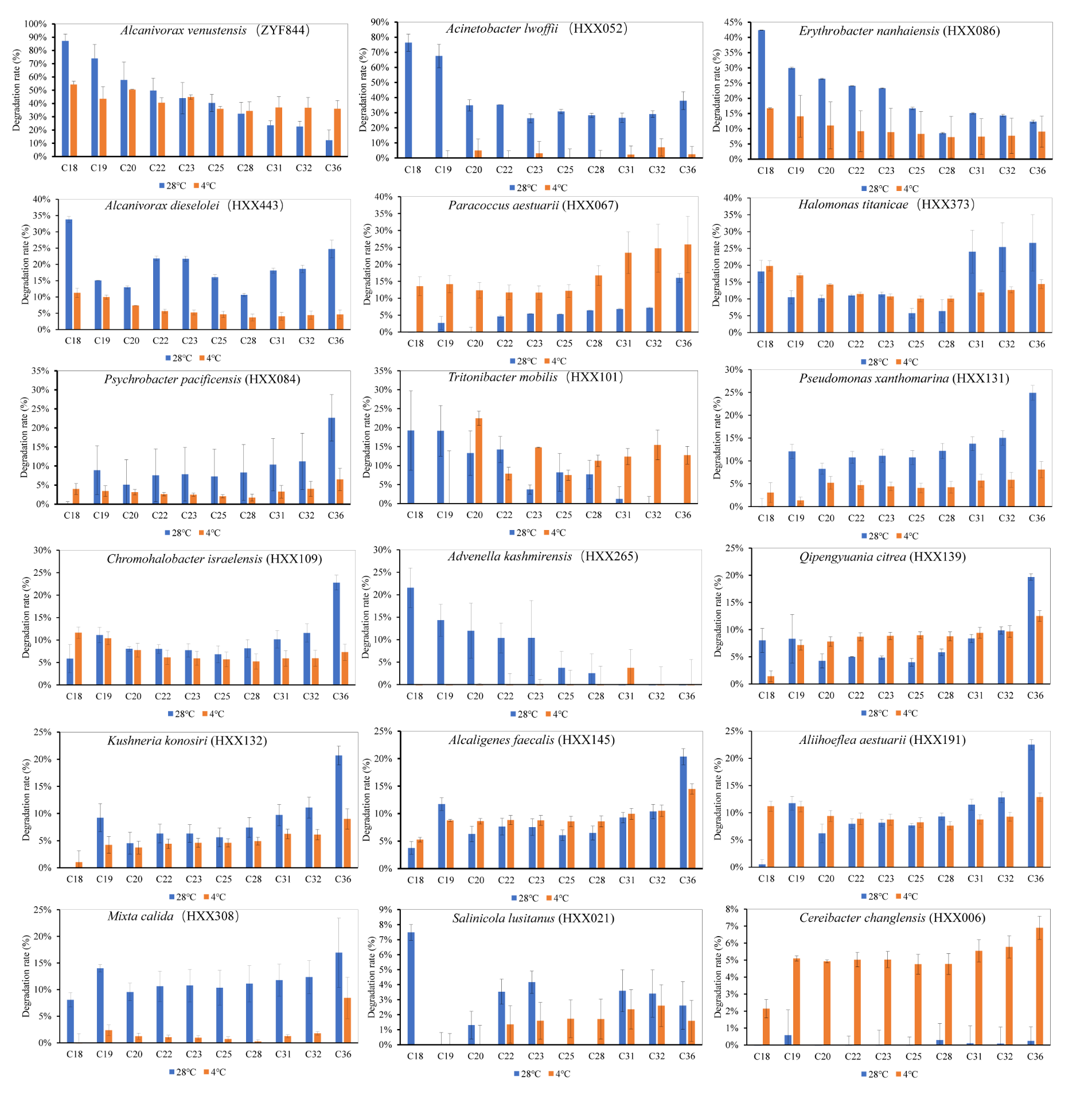
**

**Figure S2.** Determination of alkane degradation rates of representative strains from 17 cultivable *Proteobacteria* genera: comparative analysis of mixed alkanes degradation rates at different chain lengths after cultivation at 28°C for 14 days and at 4°C for 30 days. The error bars are based on triplicate measurements.


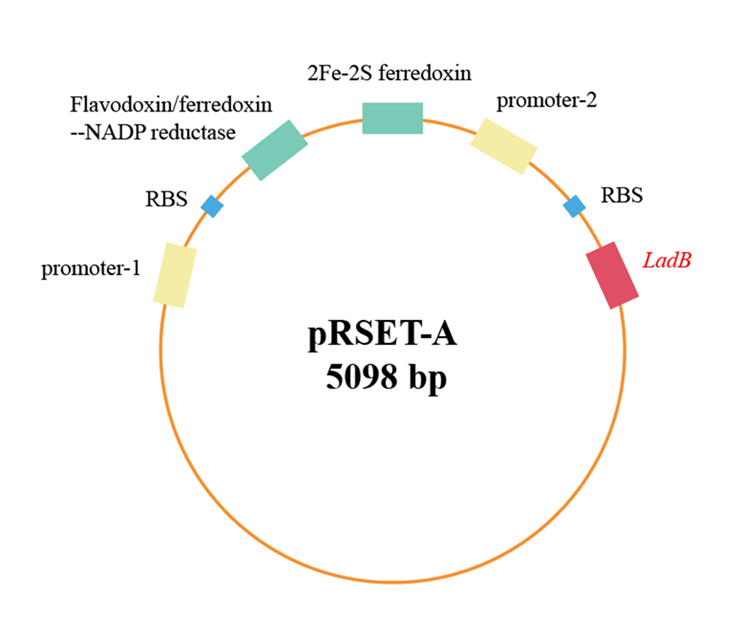


**Figure S3.** Construction diagram of recombinant expression plasmid BL21-PRSET-A-Flavodoin-2Fe-2S ferredoxin-LadB.

**
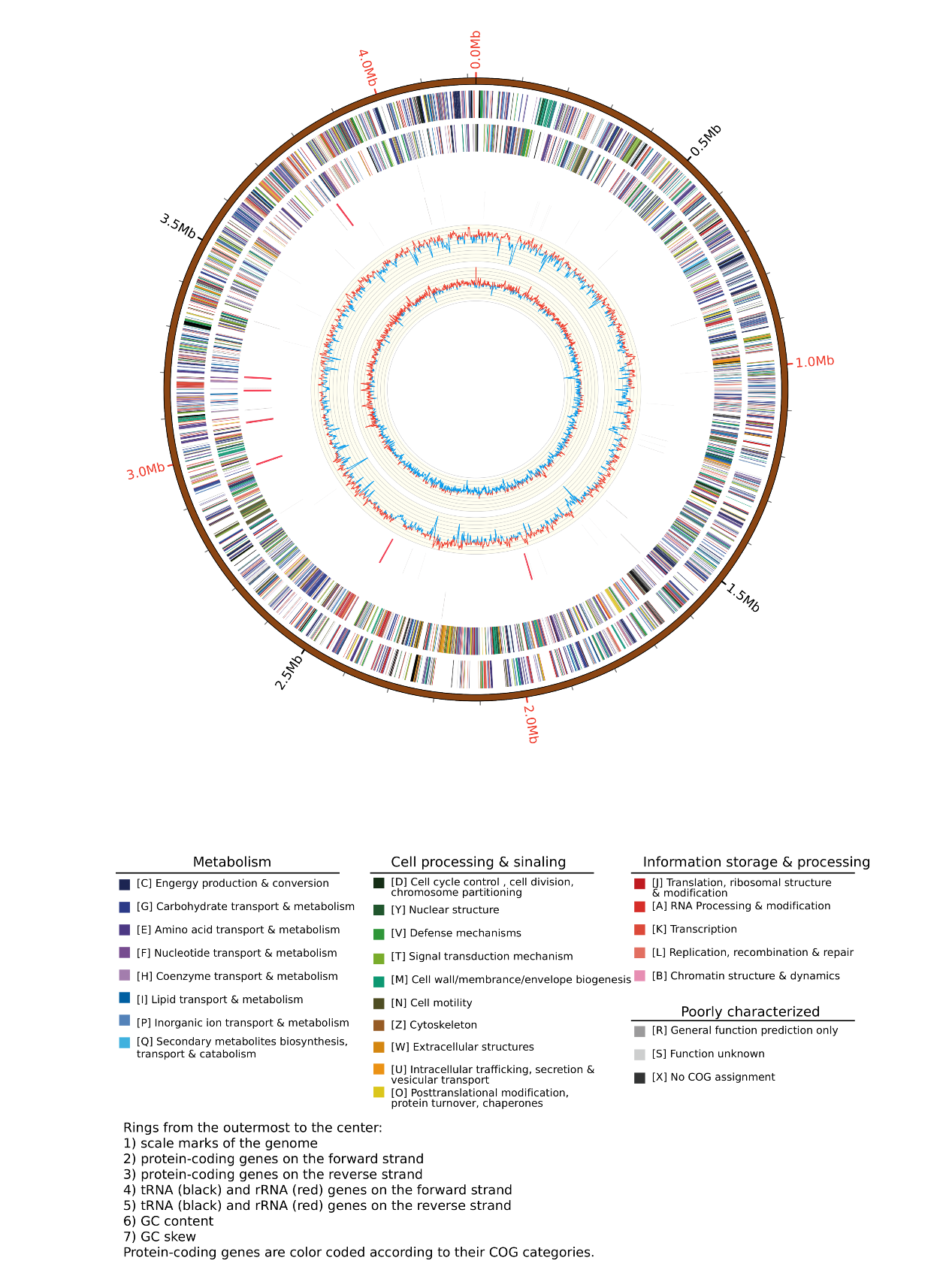
**

**Figure S4.** Circular genomic map of strain HXX308


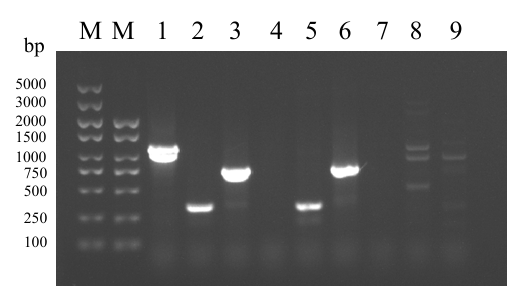


**Figure S5.** Validation of recombinant plasmids. To ensure the efficiency of expression, the flavodoxin/ferredoxin-NADP reductase and 2Fe-2S ferredoxin genes (KGOCCCKL_03163 and KGOCCCKL_04039) were inserted upstream of *LadB*, and two RBS gene were inserted before *LadB* and the flavodoxin/ferredoxin-NADP reductase and 2Fe-2S ferredoxin genes. M: marker; 1, 4, 7: PCR verification of pRSET-A-flavodoxin-2Fe-2S-ferredoxin-LadB, pRSET-A-Flavodoxin-2Fe-2S ferredoxin, and pRSET-A using the specific primers LadB-F/LadB-R, with the target band being approximately 1146 bp (LadB-F: 5'-ATGGCATTATCCGTATTC-3'; LadB-R: 5'-GCTTTGCGAGACTTTCTG-3'). 2, 5, 8: Verification using the specific primers for 2Fe-2S ferredoxin, with the target band being approximately 336 bp. 3, 6, 9: Verification using the specific primers for Flavodoxin, with the target band being approximately 747 bp.

**Table S1.** Preparation of ORN7a inorganic salt medium.

|  | **Component** | Processing method |
| --- | --- | --- |
| Solution A | NaCl，22.79 g；Na_2_SO_4_，3.98 g；KCl，0.72 g；NaBr，83.00 mg；NaHCO_3_，31.00 mg；H_3_BO_3_，27.00 mg；NaF，2.60 mg；NH_4_Cl，0.27 g；Na_2_HPO_4_·7H_2_O，89.00 mg；TAPSO，1.30 g；H_2_O，500 mL | Solutions A and B were adjusted to pH 7.6 with NaOH and then sterilized by autoclaving at 121°C for 20 min. Solution C was sterilized by filtration through a 0.22-μm pore size membrane filter. The stock solution was prepared by mixing Solutions A, B, and C. |
| Solution B | MgCl_2_·6H_2_O，11.18 g；CaCl_2_·2H_2_O，1.46 g；SrCl_2_·6H_2_O，24.00 mg；H_2_O，450 mL |  |
| Solution C | FeCl_2_·4H_2_O，2.00 mg；H_2_O，50 mL |  |

**Table S2.** Statistical analysis of sequencing data.

| Sample | Mean read length(bp) | Number of reads | Read length N50(bp) | Total bases |
| --- | --- | --- | --- | --- |
| HXX308 | 6727.84 | 225678 | 7082 | 1518326273 |

**Table S3.** Statistics of assembly results.

| Sample | Num of Contigs | Max Contig length(bp) | N50_length(bp) | Total length(bp) |
| --- | --- | --- | --- | --- |
| HXX308 | 1 | 4218089 | 4218089 | 4218089 |

**Table S4.** Statistics of coding gene prediction.

| Sample | Genome size  (bp) | Gene  number | Gene total  length (bp) | Gene average  length (bp) | Gene length / Genome (%) | Gene  density(per Kb) |
| --- | --- | --- | --- | --- | --- | --- |
| HXX308 | 4218089 | 4367 | 3626730 | 830.49 | 85.98 | 1.04 |

**Table S5.** Family type peptidase.

| Classification code | Peptidase family |
| --- | --- |
| M01 | aminopeptidase N |
| M03 | thimet oligopeptidase |
| M04 | thermolysin |
| M10 | matrix metallopeptidase-1 |
| M14 | carboxypeptidase A1 |
| M15 | zinc D-Ala-D-Ala carboxypeptidase |
| M16 | pitrilysin |
| M17 | leucyl aminopeptidase 3 |
| M19 | membrane dipeptidase |
| M20 | glutamate carboxypeptidase |
| M23 | beta-lytic metallopeptidase |
| M24 | methionyl aminopeptidase 1 |
| M28 | aminopeptidase S |
| M32 | carboxypeptidase Taq |
| M38 | isoaspartyl dipeptidase |
| M41 | FtsH peptidase |
| M42 | glutamyl aminopeptidase |
| M48 | Ste24 peptidase |
| M50 | site 2 peptidase |
| M55 | D-aminopeptidase DppA |
| M74 | murein endopeptidase |
| M78 | ImmA peptidase |
| M90 | MtfA peptidase |
| S01 | chymotrypsin A |
| S06 | IgA1-specific serine peptidase |
| S08 | subtilisin Carlsberg |
| S09 | prolyl oligopeptidase |
| S11 | D-Ala-D-Ala carboxypeptidase A |
| S12 | D-Ala-D-Ala carboxypeptidase B |
| S13 | D-Ala-D-Ala peptidase C |
| S14 | peptidase Clp |
| S16 | Lon-A peptidase |
| S24 | repressor LexA |
| S26 | signal peptidase I |
| S33 | prolyl aminopeptidase |
| S49 | signal peptide peptidase A |
| S51 | dipeptidase E |
| S54 | rhomboid-1 |
| S66 | murein tetrapeptidase LD-carboxypeptidase |
| S73 | gpO peptidase |
| S78 | prohead peptidase |

**Supplementary file 2-Supplementary Table S6-S8**

**Supplementary Table S6.** The CAZYmes annotation in the genome of strain HXX308.

**Supplementary Table S7.** The peptidases encoded by strain HXX308 annotation by MEROPS.

**Supplementary Table S8.** Genes in the mainly metabolic pathway of strain HXX308 showed in Figure 5.
